# Plasma-Exposure-Induced Mobility Enhancement of LiTFSI-Doped Spiro-OMeTAD Hole Transport Layer in Perovskite Solar Cells and Its Impact on Device Performance

**DOI:** 10.3390/ma12193142

**Published:** 2019-09-26

**Authors:** Hao Qu, Gao Zhao, Yumeng Wang, Lijuan Liang, Long Zhang, Wenya Liu, Chunmei Zhang, Chen Niu, Yi Fang, Jiazi Shi, Jiushan Cheng, Dongdong Wang

**Affiliations:** 1 Beijing Key Laboratory of Printing & Packaging Materials and Technology, Beijing Institute of Graphic Communication, Beijing 102600, China; qh18810592168@163.com (H.Q.); wangym2019@yeah.net (Y.W.); longzhang1993@foxmail.com (L.Z.); liuwenya654321@163.com (W.L.); niuchen2010@163.com (C.N.); shijiazi@bigc.edu.cn (J.S.); bjldllj@hotmail.com (L.L.); jscheng@bigc.edu.cn (J.C.); 2 Lab of Plasma Physics and Materials, School of Printing and Packaging Engineering, Beijing Institute of Graphic Communication, Beijing 102600, China; zhaohengyu369@163.com; 3 School of physics and electrical engineering, Zhengzhou Normal University, Zhengzhou 450044, China; 4 Beijing Engineering Research Center of Printed Electronics, Beijing Institute of Graphic Communication, Beijing 102600, China; fangyi@bigc.edu.cn

**Keywords:** hole mobility, spiro-OMeTAD, perovskite solar cell, plasma exposure

## Abstract

2,2′,7,7′-Tetrakis(*N*,*N*-di-p-methoxyphenyl-amine)-9,9′-spirobifluorene (spiro-OMeTAD) film currently prevails as hole transport layer (HTL) employed in perovskite solar cells (PSCs). However, the standard preparation method for spin-coated, Lithium bis(trifluoromethylsulfony) imide (LiTFSI)-doped, spiro-OMeTAD HTL depends on a time-consuming and uncontrolled oxidation process to gain desirable electrical conductivity to favor device operation. Our previous work demonstrated that ~10 s oxygen or oxygen containing gas discharge plasma exposure can oxidize spiro-OMeTAD HTL effectively and make PSCs work well. In this communication, hole-only devices are fabricated and in-situ current density-voltage measurements are performed to investigate the change in hole mobility of LiTFSI-doped spiro-OMeTAD films under plasma exposure. The results reveal that hole mobility values can be increased averagely from ~5.0 × 10^−5^ cm^2^V^−1^s^−1^ to 7.89 × 10^−4^ cm^2^V^−1^s^−1^ with 7 s O_2_ plasma exposure, and 9.33 × 10^−4^ cm^2^V^−1^s^−1^ with 9 s O_2_/Ar plasma exposure. The effects on the photovoltaic performance of complete PSC devices are examined, and optical emission spectroscopy (OES) is used for a diagnostic to explain the different exposure effects of O_2_ and O_2_/Ar plasma. High efficiency, fine controllability and good compatibility with current plasma surface cleaning techniques may make this method an important step towards the future commercialization of photovoltaic technologies employing spiro-OMeTAD hole transport material.

## 1. Introduction

The power conversion efficiencies (PCE) of photovoltaic cells, solid-state dye-sensitized solar cell (DSSC) [1,2] and recently emergent perovskite solar cell (PSC) [3,4,5,6], exhibited a remarkable increase in the last few years. Impressively, the PCE of perovskite solar cells exceeded 20% from 3.8% in less than 10 years [3,6]. One of the most important improvements contributing to higher PCEs was the introduction of a solid state hole transport layer (HTL) material, 2,2′,7,7′-Tetrakis(*N,N*-di-p-methoxyphenyl-amine)-9,9′-spirobifluorene (spiro-OMeTAD). Spiro-OMeTAD is high-soluble, high-stable (glass-transition temperature T_g_ = 121 °C) and amorphous. Because spiro-OMeTAD suffers from low hole mobility and low conductivity in its pristine form, dopant additives, typically Lithium bis(trifluoromethylsulfony)imide (LiTFSI) and tert-butyl pyridine (TBP), become necessary to improve charge carrier density, hole mobility, and energy level alignment at light absorber/HTL interface, which may increase the conductivity by 1~2 orders of magnitude, reduce the interfacial charge recombination, and thus, favor a good device operation [7,8,9]. Since LiTFSI-doped spiro-OMeTAD as HTL was developed for use in DSSC devices intentionally, it currently prevails as HTL in high-PCE PSC devices, irrespective of the mesoscopic scaffold or planar heterojunction PSC device architecture [10,11,12,13,14].

The standard spin-coated LiTFSI-doped spiro-OMeTAD HTL, commonly with chlorobenzene solvent used, exhibited high-density of nano-sized voids. These voids formed channels across the entire spiro-OMeTAD layer, which facilitated the penetration and diffusion of kinds of molecules [10,12,13,14]. Material infiltration and redistribution made the fundamental interactions complex between spiro-OMeTAD and environmental factors, such as H_2_O, O_2_, temperature, and light soaking [7,9,10,11,12,13,14]. Exposure to air, O_2_, or H_2_O substantially deteriorated the hole mobility of thermally-evaporated pristine spiro-OMeTAD film [13], while increased the hole mobility of spin-coated LiTFSI-doped spiro-OMeTAD 1~2 orders of magnitude [12]. H_2_O vapor exposure resulted in the migration and redistribution of LiTFSI and energy level shifts in spiro-OMeTAD layer [10,12]. The situation becomes even more complex because of oxidization, which is necessary to obtain appreciable conductivity of spiro-OMeTAD HTL [7,8,14,15,16,17,18]. LiTFSI is unable to directly oxidize spiro-OMeTAD, which demanded an open system to allow the entrance of O_2_ molecules. Other factors, including the concentration of Li^+^ ions and the external light intensity, also played critical roles in determining spiro-OMeTAD oxidation, and the formation of oxidized spiro-OMeTAD appeared reversible during PSCs operation in air. The difficulty in controlling the process of spiro-OMeTAD oxidation resulted in unpredictable variations and instabilities of oxidized spiro-OMeTAD concentration in HTL. Ambient conditions, under which PSCs employing spiro-OMeTAD HTL were fabricated, operated or stored, can dramatically influence the device performance, stability and reproducibility. This may be able to explain the notable variation in spiro-OMeTAD recipes used by different groups. In addition, the oxidation process under selective atmospheric conditions is commonly time-consuming, typically more than several hours. Finally, open system and uncontrolled atmospherics molecules penetration, O_2_ and H_2_O molecules in particular, may generate unwanted side effects, such as degrading the underlying active layer, and thus, deteriorating device stability.

Other than the method of using alternative dopants, e.g., Cobalt complex [2,18,19] and spiro(TFSI)_2_ [8], to chemically oxidize spiro-OMeTAD, UV-O_3_ treatment has been reported to quick enhance p-doped in spiro-OMeTAD [18]. Very recently, we have reported that oxygen or oxygen containing gas discharge plasma exposure could oxidize spiro-OMeTAD HTL effectively within seconds and made PSCs work well. The oxidation degree was analyzed qualitatively with UV-vis spectrum technique [20]. In this communication, hole-only devices were fabricated, and in-situ current density-voltage measurements were performed to investigate the real-time change in hole mobility of LiTFSI-doped spiro-OMeTAD films under plasma exposure. The hole mobility values increased averagely from ~5.0 × 10^−5^ cm^2^V^−1^s^−1^ to 7.89 × 10^−4^ cm^2^V^−1^s^−1^ with 7 s O_2_ plasma exposure, and 9.33 × 10^−4^ cm^2^V^−1^s^−1^ with 9 s O_2_/Ar plasma exposure. The effects on the photovoltaic performance of complete PSC devices are examined, and optical emission spectroscopy (OES) is used for a diagnostic to explain the different exposure effects of O_2_ and O_2_/Ar plasma. High efficiency, fine controllability and good compatibility with current plasma surface cleaning techniques may make this method an important step towards the future commercialization of photovoltaic technologies employing spiro-OMeTAD hole transport material.

## 2. Materials and Methods

### 2.1. Materials

The Fluorine-doped tin oxide (FTO) substrate with a sheet resistance of 14 Ω/square was purchased from Xiangcheng Technology Co., Ltd. (Shenzhen, China). The isopropanol (IPA), the chlorobenzene and the *N*,*N*-dimethyl formamide (DMF) solvents were purchased from Sigma-Aldrich (China) Corp. (Shanghai, China). The acid titanium dioxide solution was purchased from MaterWin New Materials Corp. (Shanghai, China). The poly(3,4-ethylenedioxythiophene):poly(styrenesulfonate) (PEDOT:PSS), the PbI_2_, the spiro-OMeTAD and the methyl ammonium iodide (MAI) were purchased from Xi’an Polymer Light Technology Corp. (Xi’an, China).

### 2.2. Device Fabrication

For perovskite planar hybrid solar cells with a structure of FTO/compact TiO_2_/Perovskite absorber/Spiro-OMeTAD HTL/Au, FTO electrodes were sequentially cleaned by ultra-sonication in acetone (15 min), IPA (15 min), ethanol (15 min) and deionized water (15 min), and then dried by N_2_ stream and treated with ultraviolet ozone (15 min). A ~30 nm-thick compact TiO_2_ electron conductor layer was deposited on the cleaned FTO substrate by spin-coating acid TiO_x_ solution at 2000 rpm for 60 s, and then heated on a hotplate at 150 °C for 10 min and at 500 °C for 30 min. A ~600 nm perovskite absorber layer was deposited onto TiO_2_/FTO substrate with a two-step method in a N_2_-filled glove box: A 0.04 mL of 460 mg/mL PbI_2_/DMF solution was spin-coated at 1200 rpm for 30 s, heated at 90 °C for 3 min, and later 0.07 mL of 10 mg/mL MAI/IPA solution was spin-coated on top of PbI_2_ complex at 6000 rpm for 60 s and heated at 120 °C for 30 min. A ~250 nm LiTFSI-Doped spiro-OMeTAD HTL was formed by spin-coating 0.075 mL of spiro-OMeTAD/chlorobenzene (70 mg HTM in 1 mL chlorobenzene) solution containing 0.07 mL of LiTFSI (170 mg LiTFSI in 1mL acetonitrile) and 0.03 mL of tbP (1 mL tert-butyl pyridine in 1 mL acetonitrile) at 2000 rpm for 30 s. Finally, a ~80 nm-thick Au electrode was deposited by thermal evaporation.

For hole-only devices to perform mobility measurement with a structure of FTO/PEDOT:PSS/Spiro-OMeTAD HTL/Au, the HTL and Au electrode were prepared by the same conditions to those used for above-mentioned perovskite solar cell device fabrications, which involved spin-coating a LiTFSI-Doped spiro-OMeTAD HTL on an FTO/PEDOT:PSS substrate, followed by thermal evaporating an Au electrode.

### 2.3. Plasma-Exposure

**Figure 1:**
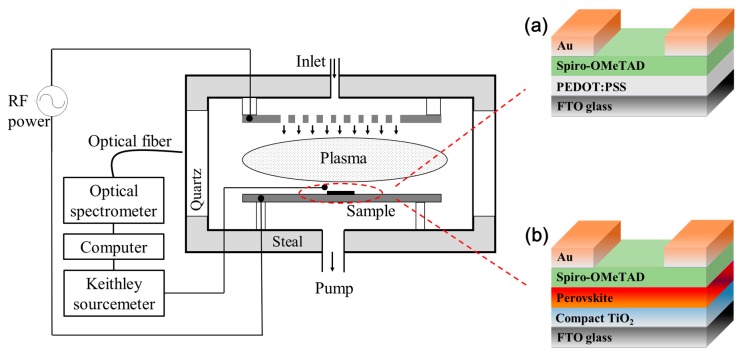
Schematics of the experimental setup used for the plasma-exposure of (**a**) hole-only device and (**b**) complete PSC device, optical emission spectrum measurement and charge mobility measurement.

A common capacitively-coupled-plasma setup consisting of two parallel electrodes was used to perform plasma exposure (Figure 1). The bottom electrode (i.e., sample holder) is grounded, while radio frequency (RF) voltage of 13.56 MHz is applied to the top electrode. The vacuum chamber was pumped down to a base pressure of 3 Pa after the hole-only or PSC devices were transferred into the chamber. Then gas was introduced into the chamber through a mass flow controller. After the pressure was stabilized, RF gas discharge plasma was generated, and the discharge time was precisely controlled by an intelligent double digital display timer. Applied power, gas flow and composition, and exposure time have been varied to ensure the desirable PSC device performance, and the optimum process conditions in our study are: For gas O_2_, O_2_ flow rate of 10 sccm, RF power of 10 W; for mixture gas O_2_/Ar, O_2_ flow rate of 5 sccm and Ar flow rate of 5 sccm, RF power of 10W. All the gases used here were 99.999% purity (PRAXAIR Corp., Beijing, China).

### 2.4. Characterizations

For the exposure-time-dependent hole-mobility measurement, hole-only devices were kept in the vacuum chamber through the entire process. For the exposure-time-evolution of PSC device performance, PSC devices were taken out and characterized in ambient conditions after exposure to plasma with specific time. As control, hole-only and PSC devices were both stored in dry air (RH 20 ± 5%) overnight (12 h) as well. The thickness of each layer was averaged from multiple measurements with a Veeco surface profiler (model Dektak 150, Plainview, NY, USA). A Keithley sourcemeter (model 4200, Cleveland, OH, USA) was used to gain the current density-voltage (*J-V*) characteristics, and an ABET solar simulator (model SUN 3000, Baltimore, MD, USA) was used to produce standard daylight (AM 1.5, 100 mW/cm^2^). The film crystallinity was analyzed by X-ray diffraction (XRD) (model D/max-2200 PC, Rigaku, Tokyo, Japan) and the absorptance properties were measured by an ultraviolet-visible spectrophotometer (model UV-2501PC, Shimadzu, Tokyo, Japan). Optical emission spectra (OES) of the gas discharge plasma were monitored by an Ocean Optics fiber spectrometer (model LAME-S-XR1-ES, Dunedin, FL, USA).

## 3. Results and Discussion

Hole-only devices (FTO/PEDOT:PSS/Spiro-OMeTAD/Au) for mobility measurement were fabricated following the published procedure [21]. The LiTFSI-doped spiro-OMeTAD HTL and above-covered gold electrode were prepared by the same conditions to those used in the complete solar cell devices (FTO/TiO_2_/Perovskite/Spiro-OMeTAD/Au). The hole-only devices were kept in the vacuum chamber, and the *J-V* characteristics were measured at intervals when plasma power was triggered off. The hole mobility values were extracted using the space charge limit current (SCLC) model, which was given by the following equation [12,21,22]:

$$J=\frac{9}{8}\varepsilon_{0}\varepsilon_{r}\mu_{h}\frac{V^{2}}{L^{3}}$$

where $\varepsilon_{0}$ is the permittivity of a vacuum, $\varepsilon_{r}$ is the dielectric constant of the spiro-OMeTAD HTL (assumed to be 3 here), $\mu_{h}$ is the hole mobility, V is voltage drop cross the device, and L is the HTL thickness.

**Figure 2:**
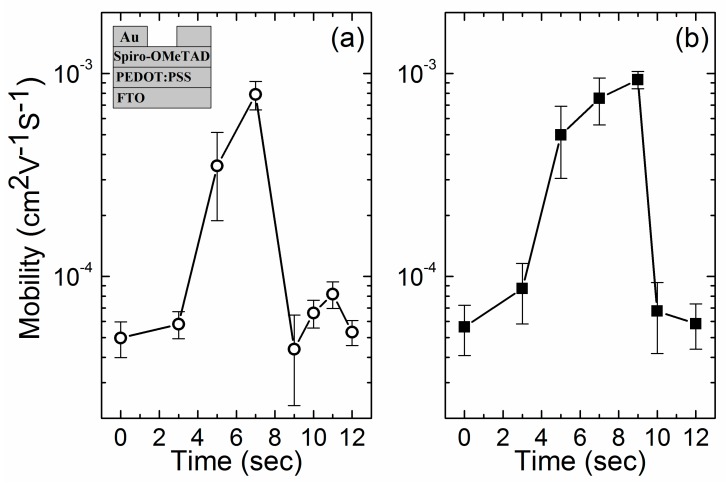
Hole mobility of LiTFSI-doped spiro-OMeTAD with different exposure time of (**a**) O_2_ plasma and (**b**) O_2_/Ar plasma. The symbols indicate the average values with the corresponding standard deviation from 4 samples. The inset shows a schematic architecture of the hole-only device for mobility measurement.

Figure 2 showed the changes in hole mobility value of LiTFSI-doped spiro-OMeTAD films which were exposed to O_2_ and O_2_/Ar plasma respectively with exposure time. Both O_2_ and O_2_/Ar plasma exposure can significantly increase the hole mobility of LiTFSI-doped spiro-OMeTAD film with very high efficiency. With 7 s O_2_ plasma exposure, the averaged mobility values increased from 4.98 × 10^−5^ cm^2^V^−1^s^−1^ (standard deviation 9.94 × 10^−6^ cm^2^V^−1^s^−1^, as-prepared HTL in a N_2_-filled glovebox) to 7.89 × 10^−4^ cm^2^V^−1^s^−1^ (standard deviation 1.26 × 10^−4^ cm^2^V^−1^s^−1^); and with 9 s O_2_/Ar plasma, the averaged mobility values can reach 9.33 × 10^−4^ cm^2^V^−1^s^−1^ (standard deviation 9.05 × 10^−5^ cm^2^V^−1^s^−1^) (Table 1). Mobility of LiTFSI-doped spiro-OMeTAD was increased by more than one order of magnitude within less than 10 s plasma exposure. The results of mobility enhancement explained our previous work which demonstrated that ~10 s plasma exposure can result in the appreciable operation of PSC devices employing LiTFSI-doped spiro-OMeTAD HTL [20]. Typically, more than several hours of exposure to H_2_O, O_2_ or dry air were necessary to functionalize LiTFSI-doped spiro-OMeTAD HTL and obtain desirable PSC device performance [10,12,14,15]. UV/O_3_ treatment was previously proposed to fast enhance the p-dope of spiro-OMeTAD, which took ~10 min to functionalize spiro-OMeTAD HTL with additional chemical oxidation of Cobalt complex dopant [18]. Up to now, plasma exposure showed the highest efficiency to oxidize and functionalize LiTFSI-doped spiro-OMeTAD HTL.

Although both O_2_ and O_2_/Ar plasma exposure could oxidize the LiTFSI-doped spiro-OMeTAD HTL and increase the hole mobility effectively and efficiently, different exposure-time-evolution behaviors were observed (Figure 2). For O_2_/Ar plasma exposure, the hole-mobility progressively increased with exposure time, reached peak value with 9 s exposure, and sharply declined to 6.75 × 10^−5^ cm^2^V^−1^s^−1^ averagely (standard deviation 2.58 × 10^−5^ cm^2^V^−1^s^−1^) with one more second exposure. It seemed that 9 s exposure was enough to fulfill the oxidation process while 10 s exposure irreversibly degraded the LiTFSI-doped spiro-OMeTAD HTL by over-oxidization. For O_2_ plasma exposure, “deterioration and recovery” behavior was observed. The hole-mobility reached peak value with 7 s exposure, declined to 4.39 × 10^−5^ cm^2^V^−1^s^−1^ averagely (standard deviation 2.07 × 10^−5^ cm^2^V^−1^s^−1^) with 9 s exposure, recovered to 8.19 × 10^−5^ cm^2^V^−1^s^−1^ averagely (standard deviation 1.22 × 10^−5^ cm^2^V^−1^s^−1^) with 11 s exposure, and degraded again to 5.32 × 10^−5^ cm^2^V^−1^s^−1^ averagely (standard deviation 7.55 × 10^−6^ cm^2^V^−1^s^−1^) with 12 s exposure.

**Figure 3:**
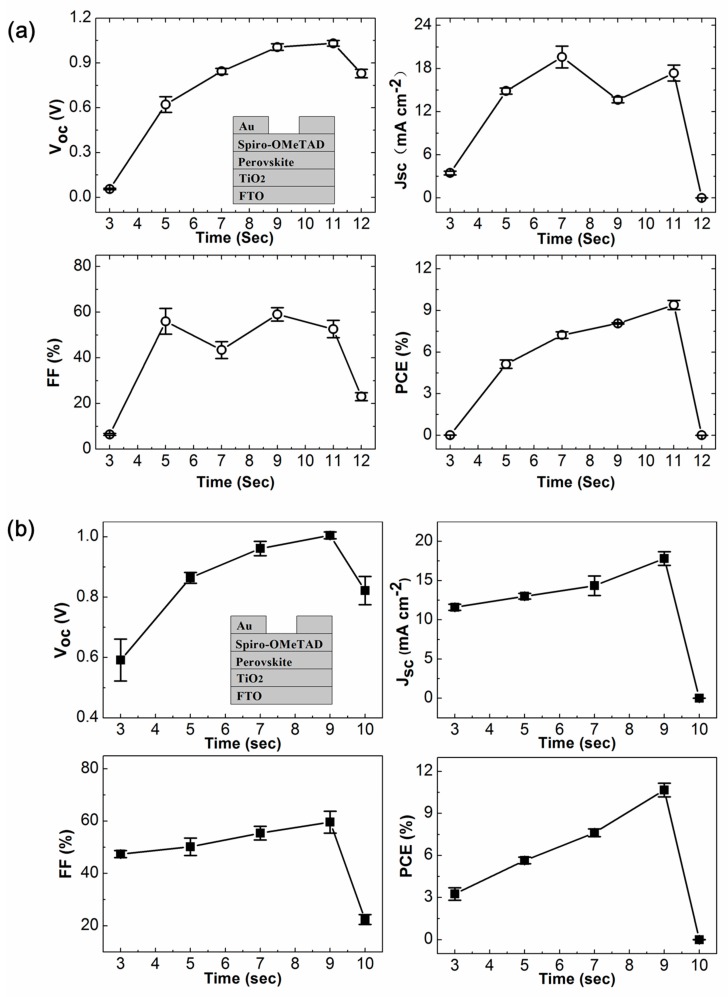
Photovoltaic parameters of the PSC devices employing LiTFSI-doped spiro-OMeTAD HTL with different exposure time of (**a**) O_2_ plasma and (**b**) O_2_/Ar plasma: Open circuit voltage *V_oc_*, short circuit current density *J_sc_*, fill factor *FF* and power conversion efficiency *PCE*. The symbols indicate the average values with the corresponding standard deviation from 5 samples. The insets show the schematic architecture of the complete PSC device.

To further confirm the effects of plasma-exposure-induced mobility enhancement, the photovoltaic performance of complete PSC devices employing LiTFSI-doped spiro-OMeTAD HTL with different exposure time of O_2_ or O_2_/Ar plasma were examined (Figure 3). For O_2_/Ar plasma exposure, all the photovoltaic parameters (open circuit voltage *V_oc_*_,_ short current density *J_sc_*, fill factor *FF* and power conversion efficiency *PCE)* increased progressively over exposure time and reached maximum values at an exposure time of 9 s, with an average *V_oc_* = 1.00 V (standard deviation 0.01 V), an average *J_sc_* = 17.82 mA (standard deviation 0.87 mA), an average *FF* = 59.6% (standard deviation 4.16%) and an average *PCE* = 10.76% (standard deviation 0.48%). It’s extraordinary that with one more second (total 10 s) of O_2_/Ar exposure, while the photovoltage decreased a little and remained at *V_oc_* = 0.82 V (standard deviation 0.05 V), the photocurrent was deteriorated so severely to almost zero which directly resulted in the failure of PSC device with a *PCE <* 0.01%. These results of photovoltaic performance evolution from complete PSC devices were strictly consistent with the outcomes of hole-mobility changement from hole-only devices. Similar over-oxidation of LiTFSI-doped spiro-OMeTAD HTL and deterioration of PSC performance were reported when increasing the UV/O_3_ treatment time to ~20 min [18]. The improvement of device performance with proper plasma exposure can be understood, since plasma-exposure-induced mobility enhancement of LiTFSI-doped spiro-OMeTAD HTL will substantially decrease the device serial resistance (*R*_s_), which has a pronounced effect on the fill factor, and favor the carrier collection efficiency across the perovskite/spiro-OMeTAD interface. However, for O_2_ plasma exposure, some unexpected behaviors were observed. The higher mobility of spiro-OMeTAD HTL did not always lead to better device performance. As shown in Figure 3a, when the hole-mobility reached peak value with 7 s exposure, the device exhibited maximum short current density *J_sc_* = 19.61 mA (standard deviation 1.52 mA), but significantly deteriorated fill factor *FF* from 56% (5 s exposure, standard deviation 5.66%) to 43.4% (standard deviation 3.65%) averagely; 9 s exposure resulted in remarkably decreased hole mobility and short current density *J_sc_* = 13.63 mA (standard deviation 0.42 mA), but recovered fill factor *FF* = 59% (standard deviation 2.92%) and better power conversion efficiency *PCE* = 8.07% (standard deviation 0.04%); 11 s exposure resulted in recovery of hole mobility and short current density *J_sc_* = 17.36 mA (standard deviation 1.1 mA), a little decreased fill factor *FF* = 52.6% (standard deviation 3.78%), but best power conversion efficiency *PCE* = 9.4% (standard deviation 0.33%); further longer plasma exposure dramatically failed the PSC device finally. Exposed to O_2_ plasma longer than 12 s or O_2_/Ar plasma longer than 10 s, the hole mobility values of LiTFSI-doped spiro-OMeTAD kept ~5.0 × 10^−5^ cm^2^V^−1^s^−1^, and the PSC devices remained no efficiency. We had tried longer exposure time until 60 s, and noticeable changes were not observed anymore.

**Figure 4:**
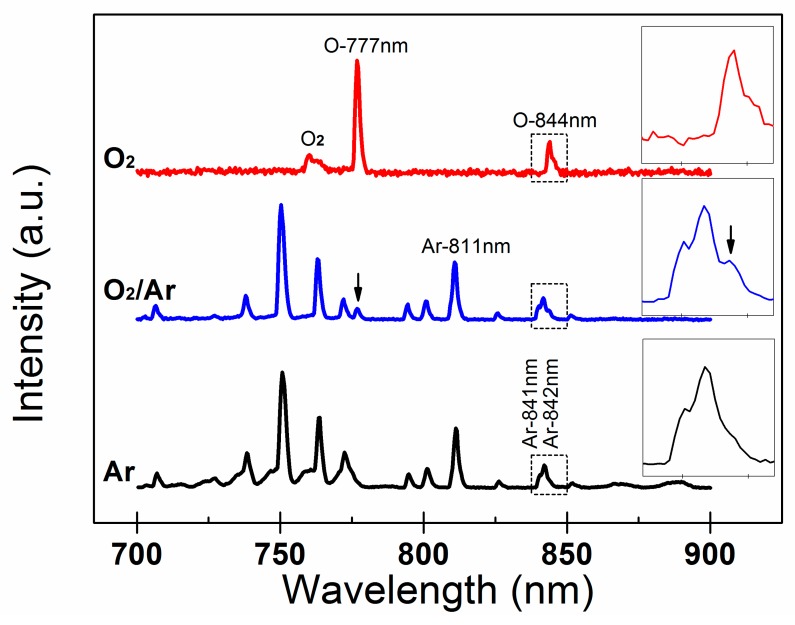
Optical Emission spectra of the Ar (black line), O_2_/Ar (blue line) and O_2_ plasma (red line). The insets show the enlarged part from 837 nm to 847 nm, and the two peaks other than O-844 nm are Ar spectral lines, Ar-841 nm and Ar-842 nm, respectively.

**Figure 5:**
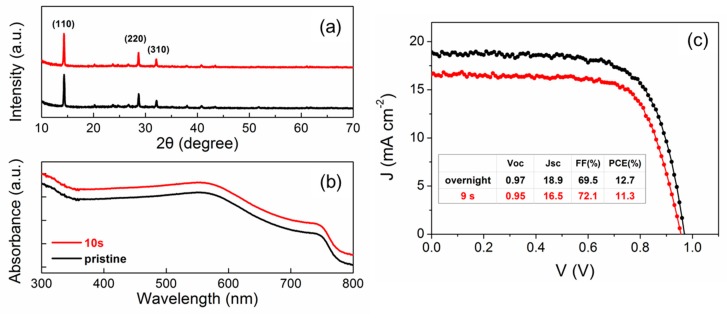
(**a**) XRD patterns of the perovskite films as-prepared in N_2_-filled glovebox (black) and exposed to O_2_/Ar plasma for 10 s (red); (**b**) UV-vis absorbance spectra of the perovskite films as-prepared in N_2_-filled glovebox (black) and exposed to O_2_/Ar plasma for 10 s (red); (**c**) Current density-Voltage characteristics of perovskite solar cells stored in dry air overnight (12 h) and exposed to O_2_/Ar plasma for 9 s.

Atomic oxygen radical has been recognized as the most reactive specie in oxygen plasma, and here optical emission spectroscopy (OES) was used for a diagnostic [23,24,25,26]. Figure 4 showed the OES spectra of O_2,_ O_2_/Ar and Ar plasma. In the displayed range from 700 to 900 nm, the spectrum of O_2_ plasma was dominated by the two atomic oxygen lines at 777 and 844 nm. O-777 nm emission corresponding to O $\left(3p^{5}P\to 3s^{5}S\right)$ transition is mainly generated by the dissociative excitation between electrons and oxygen molecules ($e+O_{2}\to O^{*}+O+e)$, while O-844 nm emission corresponding to O $\left(3p^{3}P\to 3s^{3}S\right)$ transition dominantly came from the direct atomic excitation between electrons and oxygen atoms $\left(e+O\to O^{*}+e\right)$ [23]. The oxygen atmospheric band (O_2_-*A* band) at 760 nm corresponding to O_2_ molecule transition $\left(b^{1}\sum_{g}^{+}-X^{3}\sum_{g}^{-}\right)$ was also observed [27,28]. For O_2_/Ar plasma, two atomic oxygen lines, O-777 nm and O-844 nm (denoted by two arrows), were newly present compared with Ar plasma. Ar-811 emission corresponding to Ar $\left(2p_{9}\to 1s_{5}\right)$ transition is the spectrum line of interest, which is related to argon metastable states [24,25,26]. Oxygen radicals, particularly atomic oxygen, are assumed to be mainly responsible for the oxidation process. It is worth mentioning that emitting intensity depends on the population of the relevant excited states and not directly on the density of atomic oxygen in the fundamental state. To measure the absolute atomic oxygen density, space- and time-resolved distribution, other techniques such as two-photon absorption laser-induced fluorescence spectroscopy had to be employed. Based on the OES results, here we try to propose a preliminary mechanism to explain the effects of the addition of Ar to O_2_ plasma. For O_2_/Ar plasma, a portion of the Ar was excited to the metastable state ($Ar^{M}$) which played a critical role in increasing the electron temperature, the plasma density, and the degree of oxygen molecular dissociation. Atomic oxygen density can be significantly increased via quenching of argon metastables ($Ar^{M}+O_{2}\to Ar+O+O)$ [26]. On the other hand, Ar was chemically inert. No noticeable oxidation and mobility enhancement were observed for the LiTFSI-doped spiro-OMeTAD HTL exposed to Ar plasma [20]. In addition, noticeable surface/structure modification, such as sputter etching was hardly observed under our low-energy plasma process within several seconds either (Figure 5), which was also demonstrated in another very recent work [29]. Atomic oxygen radicals diluted in inert Ar atmosphere facilitated a mild and uniform interaction with spiro-OMeTAD. As far as O_2_ plasma was concerned, it was chemically reactive. Chemical etching, also called plasma ashing, may remove organic matter and enlarge the size of nano-sized voids in spiro-OMeTAD HTL. The diffused negative ions from perovskite surface, such as I^−^ into the voids in spiro-OMeTAD HTL may prevent oxidation of spiro-OMeTAD, and degrade the electric properties [30,31], which led to the “deterioration and recovery” behavior of spiro-OMeTAD HTL exposed to O_2_ plasma.

## 4. Conclusions

In summary, the plasma-exposure-induced mobility enhancement of LiTFSI-doped spiro-OMeTAD hole transport layer in perovskite solar cells and its impact on device performance was investigated in this communication. Between O_2_ and O_2_/Ar plasmas, we confirmed that O_2_/Ar plasma exposure offered superior process results: 9 s O_2_/Ar plasma exposure can increase the hole mobility from ~6 × 10^−5^ to 1 × 10^−3^ cm^2^V^–1^s^–1^, and make the PSC devices reach ~90% performance of the ones stored in dry air overnight (Figure 5). The ~10% discrepancy may result from the influence of H_2_O molecules, which can favor both the conductivity of spiro-OMeTAD HTL and recrystallization of perovskite active layer in hours, not seconds [14,15]. OES was used as a diagnostic tool to explain the “deterioration and recovery” behaviors of hole mobility under O_2_ plasma exposure preliminary. High efficiency, fine controllability and good compatibility with current plasma surface cleaning technology and instruments may help us to better understand the fundamental working mechanism of spiro-OMeTAD HTL in PSC devices, and contribute significantly to the future commercialization of photovoltaic technologies using spiro-OMeTAD hole transport material.

## Figures and Tables

**Table 1:** Average hole-mobility values of LiTFSI-Doped spiro-OMeTAD film exposed to plasma for different time and stored dark in dry air overnight (12 h).

Time	Mobility [cm^2^V^−1^s^−1^] (O_2_)	Mobility [cm^2^V^−1^s^−1^] (O_2_/Ar)
0 s	4.98 × 10^−5^	5.64 × 10^−5^
3 s	5.82 × 10^−5^	8.72 × 10^−5^
5 s	3.51 × 10^−4^	4.98 × 10^−4^
7 s	7.89 × 10^−4^	7.55 × 10^−4^
9 s	4.39 × 10^−5^	9.33 × 10^−4^
10 s	6.61 × 10^−5^	6.75 × 10^−5^
11 s	8.19 × 10^−5^	
12 s	5.32 × 10^−5^	5.85 × 10^−5^
* 12 h	6.87 × 10^−4^	

* Stored dark in dry air overnight (12 h) is illustrated here as a reference.
